# Intensity and Stationarity Analysis of Land Use Change Based on CART Algorithm

**DOI:** 10.1038/s41598-019-48586-3

**Published:** 2019-08-22

**Authors:** Xiao Sang, Qiaozhen Guo, Xiaoxu Wu, Ying Fu, Tongyao Xie, Chengwei He, Jinlong Zang

**Affiliations:** 1 School of Geology and Geomatics, Tianjin Chengjian University, Tianjin, 300384 China; 2 State Key Laboratory of Remote Sensing Science, College of Global Change and Earth System Science, Beijing Normal University, Beijing, 100875 China

**Keywords:** Ecological modelling, Sustainability

## Abstract

Land use directly reflects degree of human development and utilization of land. Intensity analysis of land use is a quantitative method to analyze land use changes. In this paper, land use changes in Tianjin were studied using Thematic Mapper (TM) remote sensing images in 1995, 2000, 2005, 2010 and Operational Land Imager (OLI) remote sensing image in 2015. Land use information was extracted using decision tree classification method based on CART (Classification and Regression Trees) algorithm. This paper introduced land use intensity analysis to analyze its change intensity and stationarity, respectively at interval, category and transition levels. Based on the theory, new models were developed in the transition level to analyze land use change pattern. The analysis quantifies the contribution of a certain land categories to land use change during a specific time interval. The change of land use during 1995–2015 indicated that Tianjin experienced rapid urban development with the area of urban land increased by about 7.5%. This study provided a reference for the sustainable development of land use in Tianjin.

## Introduction

Land use intuitively records surface features which are changed by human activities to satisfy the needs for natural resources. Land use change has a major impact on environmental problems such as land degradation, global climate change and biodiversity by urban expansion, forest overexploitation, and agricultural intensification^1–4^. Studies in this field improve the understanding of local development and provide a basis for sustainable development planning.

The common data used in land use change analysis include Landsat image^5–9^, MODIS image^10–16^, high-resolution remote sensing images with meter grade^17–19^, radar image^20–23^, and some other data like satellite scatterometer data and thematic data^24–26^. Research on land use could help us to optimize land development and utilization. In land use research, many methods have been proposed to improve accuracy of land use classification^27–32^. CART (Classification and Regression Trees) algorithm is one of the decision tree classification algorithms widely used in land use classification^33,34^. CART algorithm constructs a binary tree using a randomly selected sample of remote sensing, and then uses the tested sample to prune. Though many machine learning algorithms have been applied like SVM (Support Vector Machine)^35,36^ and RF (Random Forest)^37,38^, one study pointed out that advanced classification algorithms may not always have advantages when they were applied to proceed multispectral image data^39^.

In terms of land use change analysis, some studies focused on the characteristics of land use change without systematic analysis, like the impact of conservation status and ownership on land use and land cover change (LULC)^40^. Since the concept of intensity analysis was put forward^41^, it becomes an important research aspect in land use change. Pontius *et al*.^42^ pointed out problems that can be solved using intensity analysis in the field of land use research. Some research analyzed land use change using intensity analysis in many study areas, like southern coastal areas of China^43^, and Parnitha and Penteli in Greece^44^. Intensity analysis has a top-down hierarchy at interval, category and transition levels and a layered progressive system that demonstrates the process of land use change. At the same time, the intensity analysis evaluates the process of land use change by setting the stability. Stable land use conversion may not necessarily lead to sustainable development, but it provides an important basis for sustainable development. When the stable development of a certain land use has a favorable effect on the surrounding or internal factors, this state can be continued in a short time in the future, but it needs to be within the carrying capacity of the environment; if it adversely affects the environment or internal factors, it should delay or even hinder its development through appropriate measures, leaving a space for development of favorable land use transformation. Even though the intensity analysis can be used to analyze land use more systematically, it could not completely reflect its change. In this paper, new models were developed to quantify the contribution of land categories to land use change, and taking Tianjin as the study area we analyzed land use change during 1995–2015 based on the intensity analysis to provide reference for urban sustainable development for Tianjin.

## Results

**Figure 1:**
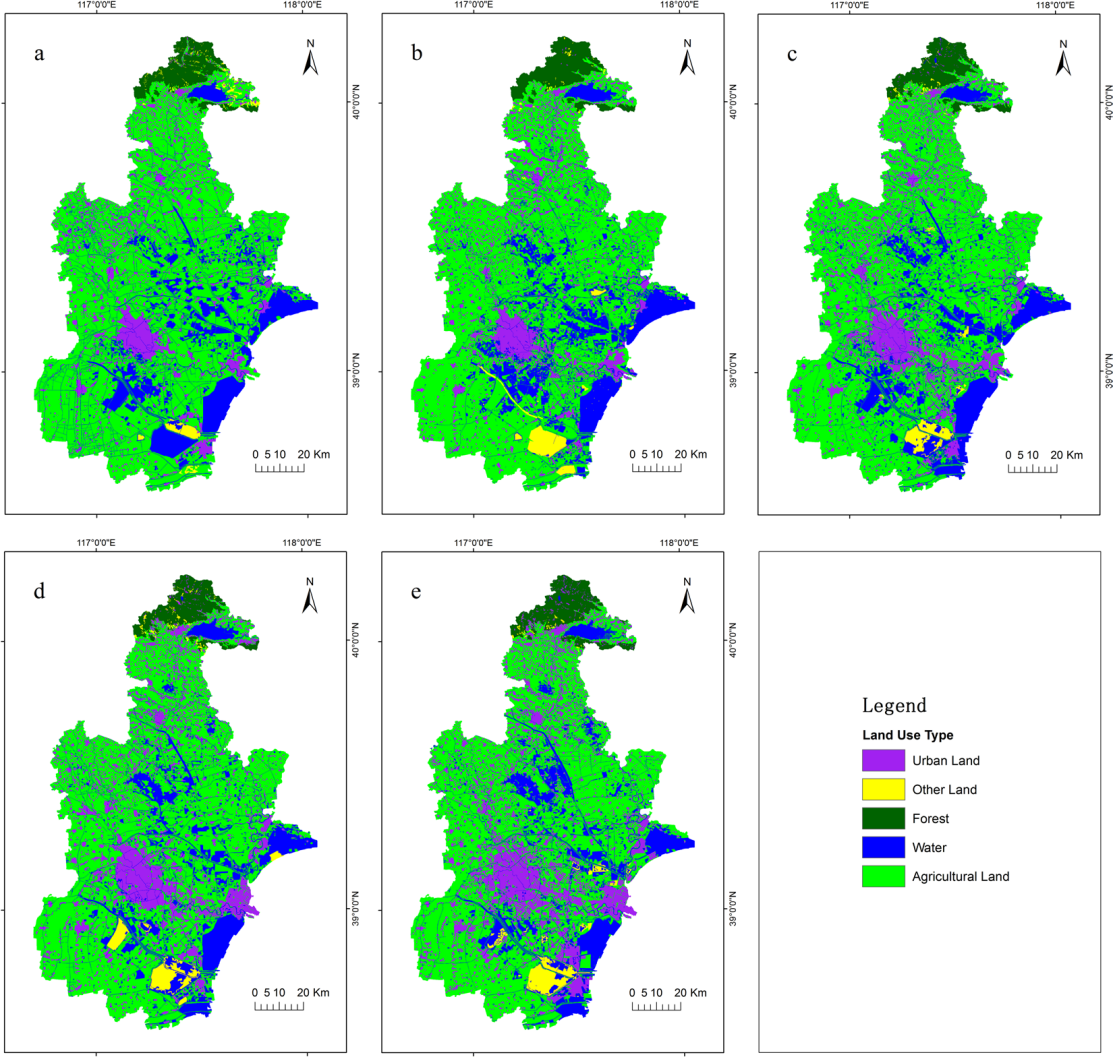
Land use classification maps of Tianjin, (**a**) 1995, (**b**) 2000, (**c**) 2005, (**d**) 2010 and (**e**) 2015.

Land use classification accuracy of Tianjin was listed in Tables 1 and 2. The relationship between kappa coefficient and classification quality was shown in Table 3 ^45^. Land use classification maps of five years were shown in Fig. 1. According to Tables 1–3 and Fig. 1, classification results meet accuracy requirement of the data.

**Table 1:** Land use classification accuracy of Tianjin respectively for producers and users.

Year		Water	Forest	Urban Land	Agricultural Land	Other Land
1995	Producers/%	95.74	90.91	92.31	96.61	25.00
	Users/%	93.75	100.00	92.31	95.15	100.00
2000	Producers/%	91.30	100.00	96.88	98.73	71.43
	Users/%	97.67	100.00	100.00	95.68	83.33
2005	Producers/%	87.18	100.00	94.12	98.75	75.00
	Users/%	100.00	100.00	94.12	95.18	100.00
2010	Producers/%	80.65	87.50	75.00	97.74	57.14
	Users/%	96.15	100.00	90.91	84.97	80.00
2015	Producers/%	72.22	85.71	87.04	97.83	100.00
	Users/%	96.30	100.00	94.00	88.82	88.89

**Table 2:** Land use classification accuracy of Tianjin.

Classification accuracy	1995	2000	2005	2010	2015
Overall Classification Accuracy/%	94.80	96.40	96.00	88.40	91.20
Kappa Coefficient	0.90	0.93	0.92	0.81	0.85

**Table 3:** Kappa Coefficient statistics and classification quality.

Kappa Coefficient	Classification quality
<0.00	very poor
0.00~0.20	poor
0.20~0.40	general
0.40~0.60	good
0.60~0.80	great
0.80~1.00	excellent

The total area of Tianjin was estimated 11,511.02 km^2^. Table 4 showed area size of each land category and their proportion in 1995, 2000, 2005, 2010 and 2015 respectively. Generally, agricultural land was the main land use category, followed by water and urban land; area of these three land categories accounted for about 95% of the total area in Tianjin. During the study period, the categories with the greatest change were urban and agricultural land with 7.5% increase and 7.5% decrease, respectively. And water area decreased about 1.35%, other land area increased about 1.09%, forest had a slight increase in area. Throughout the study period, the area of urban land continued to increase and the area of agricultural land continued to decrease. The area of water changes to forest and other land was fluctuating. The main transition was from agricultural land to urban land and which indirectly indicated that Tianjin experienced rapid urban development.

**Table 4:** Area size and proportion of land categories of Tianjin.

Year		Water	Forest	Urban Land	Agricultural Land	Other Land
1995	area/km^2^	1943.80	449.78	1265.16	7739.39	112.90
	proportion/%	16.89	3.91	10.99	67.23	0.98
2000	area/km^2^	1928.53	475.25	1322.45	7570.41	214.39
	proportion/%	16.75	4.13	11.49	65.77	1.86
2005	area/km^2^	1960.52	454.04	1547.16	7389.78	159.52
	proportion/%	17.03	3.94	13.44	64.20	1.39
2010	area/km^2^	1809.72	450.53	1842.40	7172.70	235.67
	proportion/%	15.72	3.91	16.01	62.31	2.05
2015	area/km^2^	1788.81	472.20	2128.62	6883.39	238.01
	proportion/%	15.54	4.10	18.49	59.80	2.07

### Land use intensity analysis at interval level

**Figure 2:**
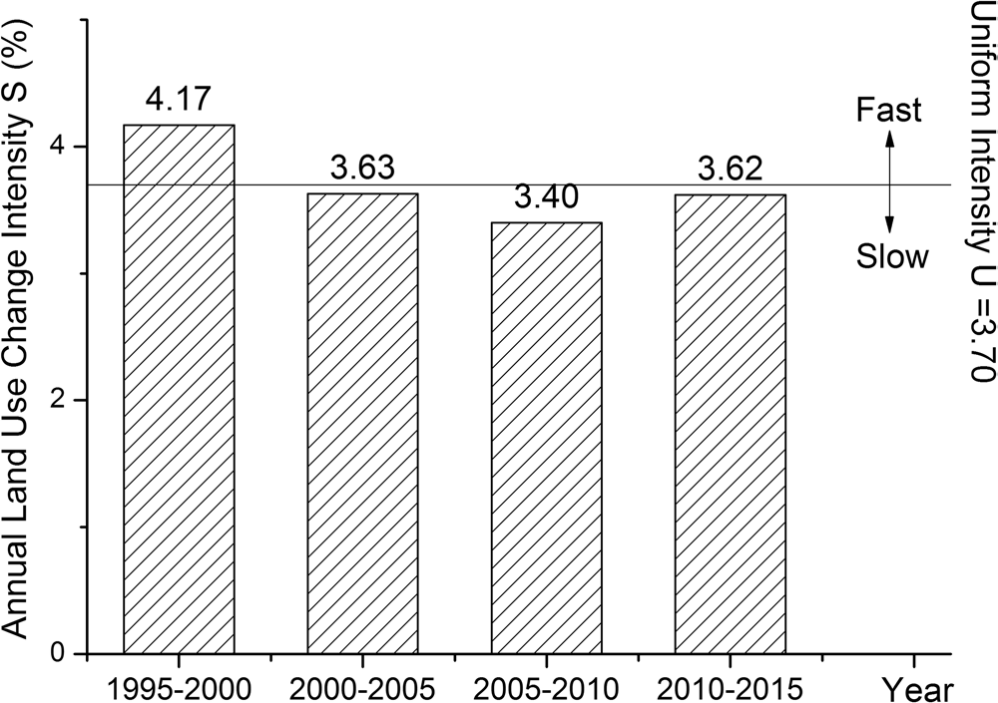
Land use intensity change of Tianjin at interval level.

From 1995 to 2000 (Fig. 2), annual land use change intensity of 4.17% was greater than the uniform intensity (3.70%), which indicated that land use change was fast during this period. But from 2000 to 2005, from 2005 to 2010 and from 2010 to 2015, annual land use change intensity were respectively 3.63%, 3.40% and 3.62%, all of which were smaller than the average value; this indicated land use change was slow during the three periods. In 1994, State Department of China issued Decision of the State Council on Deepening the Reform of Urban Housing System (National issued [1994] Num.43)^46^ and then Tianjin government approved Deepening the Housing System Reform Program Implementation Details in Tianjin^47^ that carried out on March 15, 1995. So with the implementation of these policies, the dramatic change of land use took place from 1995 to 2000.

### Land use intensity analysis at category level

**Figure 3:**
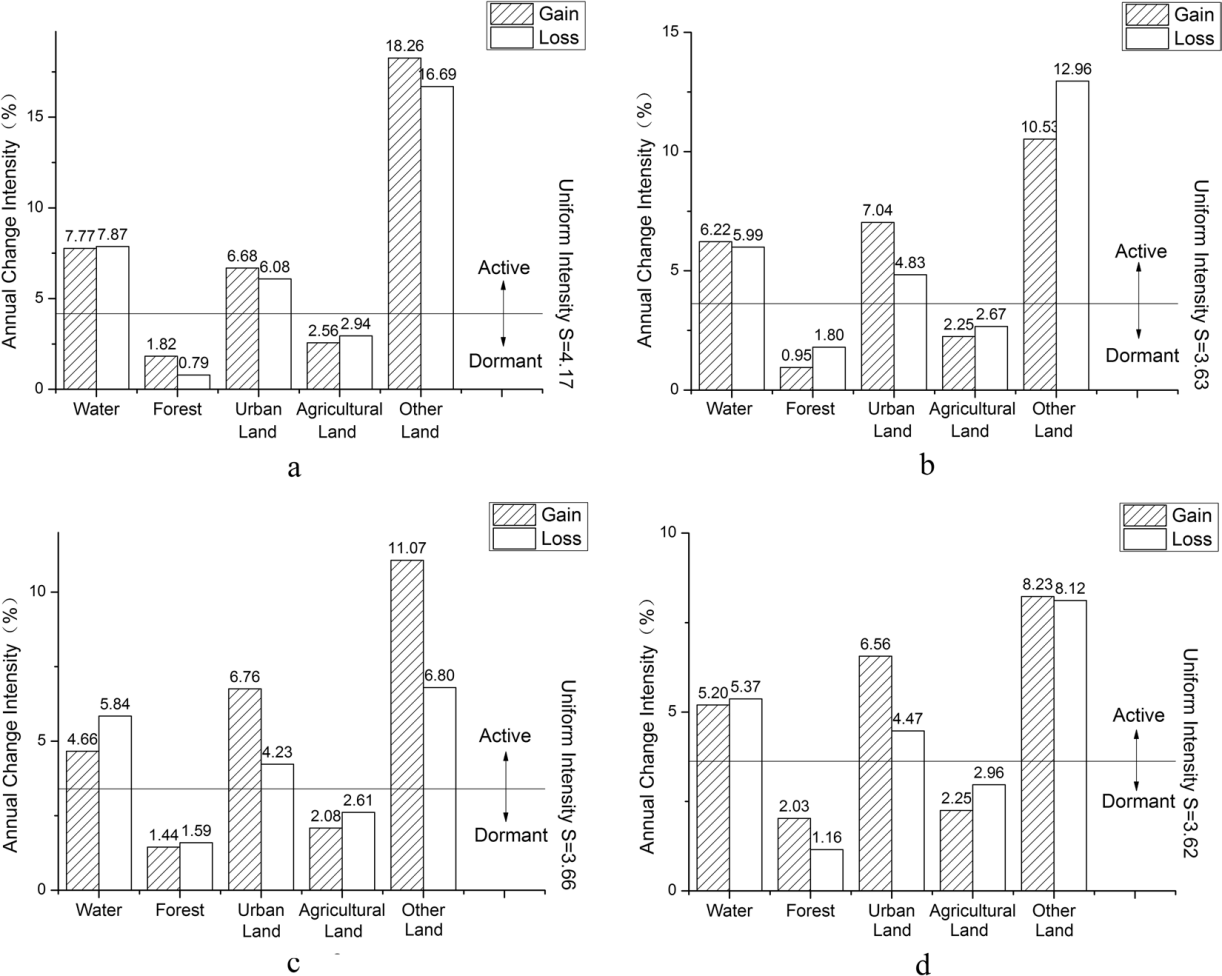
Land use intensity change at category level of Tianjin. (**a**) 1995–2000, (**b**) 2000–2005, (**c**) 2005–2010; and (**d**) 2010–2015.

According to land use intensity change of Tianjin at category level (Fig. 3), the most active change was observed for other land. Area of other land occupied less than 3% of total area of Tianjin. Expansion of the city affected change of other land. The change intensity of water was active due to utilization of water area. The change intensity of urban land was also greater than the average intensity. Over the past 20 years, rapid economic development had attracted a large number of immigrant people to Tianjin; and under influence of national policy and planning, area of urban land showed an increasing trend. Agricultural land accounted for more than 60% of total area of Tianjin. Even if its change intensity was relatively flat, its change area was still considerable. With popularization of the concept of sustainable development and implementation of environmental protection and other policies, change of forest was more stable and weak.

### Land use intensity analysis at transition level

**Figure 4:**
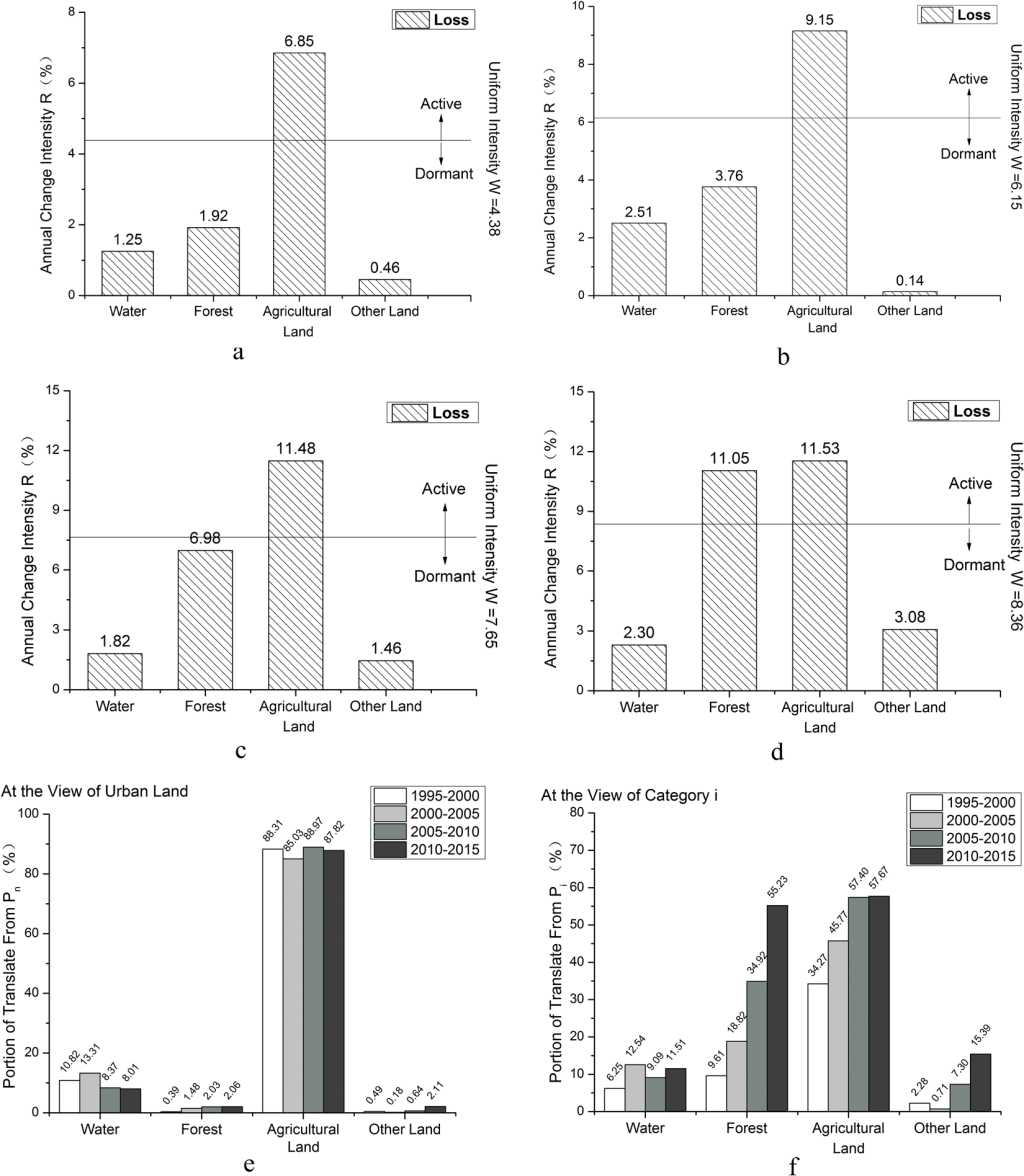
Intensity change of urban land based on gains while other categories based on losses at transition level in Tianjin in four time intervals. (**a**) 1995–2000; (**b**) 2000–2005; (**c**) 2005–2010; (**d**) 2010–2015; (**e**) intensity change of urban land from water, forest, agricultural land and other land in a particular time interval from perspective of increased area of urban land; (**f**) change intensity of urban land from water, forest, agricultural land and other land in a particular time interval from perspective of water, forest, agricultural land and other land respectively.

As for urban land, the overall change trend was increasing. According to intensity change of urban land at transition level in Tianjin during 1995–2015 (Fig. 4a–d), the transitions of urban land were mainly from agricultural land and the uniform intensity W was on the rising trend. As for water, in 1994, resolution on establishment of the Binhai New District was passed in the second meeting of 12^th^ session of People’s Congress in Tianjin. During the “Eleventh Five-Year Plan” period (2006~2010), development and opening of the Tianjin Binhai New Area had been rapidly promoted. This further advanced economic development in Tianjin, which also contributed to development and utilization of waters. As for forest (Fig. 4a–d), development of tourism industry in Pan Mountain promoted its transition to urban land, area of which had continued rising, but the transition intensity was not great due to environmental protection. As for agricultural land (Fig. 4a–d), the annual change intensity were all above the uniform intensity and the W value became bigger and bigger. As for other land (Fig. 4a–d), its transition intensity was largest during 2010–2015 and lowest during 2000–2005. For the four periods, all the transitions from agricultural land to urban land were active, so was the transition from forest to urban land during 2010–2015. And the others were dormant. Among sources of transition to urban land (Fig. 4e), agricultural land was the main source, followed by water, and then by forest and other land. According to Fig. 4f, as for the transition to urban land, transition intensity from agricultural land occupied the most but it accounted for no more than 60% of the areas transformed from agricultural land; this transition intensity accounted for 34.27% during 1995–2000, and then increased annually, and finally stably reached at about 57%. The transition of forest to urban land had increased annually. The transition intensity of water to urban land fluctuated at around 10%. The transition intensity of other land to urban land was least during 2000–2005 and then increased radically to 15.39% during 2010–2015.

**Figure 5:**
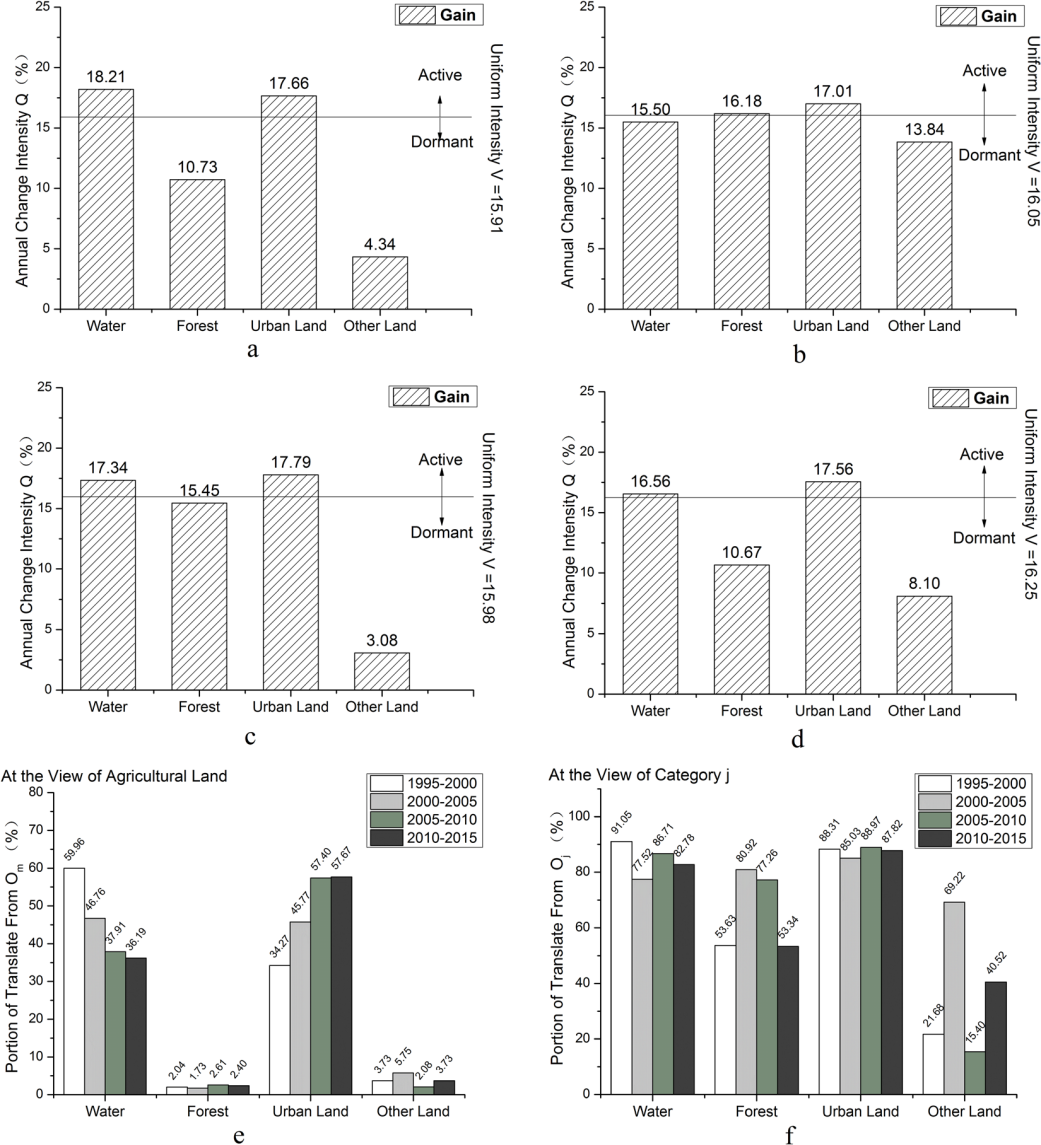
Intensity change of agricultural land based on losses while other categories based on gains at transition level in Tianjin in four time intervals. (**a**) 1995–2000; (**b**) 2000–2005; (**c**) 2005–2010; (**d**) 2010–2015; (**e**) change intensity from agricultural land to water, forest, urban land and other land in a particular time interval from perspective of increased agricultural land; (**f**) change intensity from agricultural land to water, forest, urban land and other land in a particular time interval from perspective of water, forest, urban land and other land.

As for agricultural land, the overall change trend was decreasing. Transition direction for agricultural land was mainly to urban land and water (Fig. 5a–d). Due to rapid economic development, urban land showed an expansion trend with its main source from agricultural land. According to the image, a certain proportion of agricultural land was transformed to water because of rainstorm. Due to the policy of returning agricultural land to forest, a slight proportion of agricultural land was transformed to forest which accounts for about 2% of the total transformed area of agricultural land. For the four periods, all the transition from agricultural land to urban land were active and transition from agricultural land to other land were dormant. Meanwhile, transitions from agricultural land to water were dormant during 2000–2005 and active at other periods. Transitions from agricultural land to forest were active during 2000–2005 and dormant at other periods. Transition of agricultural land to urban land and water had a high intensity but with low intensity to forest and other land (Fig. 5e). Agricultural land was main sources for area increase of urban land and water, even for forest in some periods (Fig. 5f). Transition proportion from agricultural land to other land was small during 1995–2000 and 2005–2010, and large during 2000–2005 and 2010–2015.

## Discussion

The change of land use is staged, and the transition of stable land use categories is a process from quantitative change to qualitative change. In the stage of quantitative change, a steady increase or a steady decrease, accumulated over a long period of time, will have an irreversible effect on the final state. Therefore, the stable transition of land use categories over a long period of time can be used as a basis for the relevant departments to predict the land use structure in the future, or to estimate the extent to which land use can be changed. Some land use categories will have favorable or unfavorable effects on the surrounding environment during the process of change. The stable transition trend of land use is the basis for some effects. In the sense of sustainable development, stable land use transition provides clearer information than unstable transition. Stable land use transition reflects the regularity of long-term land use and can be used for predicting initial land use change in the future; while the unstable transformation is difficult to determine the general trend of land use developmentso that it can’t provide a clear reference for the sustainable development of land use. Based on that, understanding change of land use contributes to the realization of sustainable development.

Sustainable development is the development that meets the needs of the present society without compromising the ability of future generations to meet their own needs^48^. Tianjin, as a municipality with rapid economic development, attracts people from all over the country. Rapid population growth has increased the demand for natural resources especially land. According to the results of this study, the transition of agricultural land to urban land was stable during 1995–2015. This means that in the short term, the agricultural land will continue to decrease and the urban land will continue to increase. The sustainable development goals proposed by the United Nations include education, infrastructure, health lives, and climate^49^. Undoubtedly, the increased urban land meets the basic living requirement of Tianjin residents’ education and infrastructure. But it also had effect to climate in a certain degree. From 1995 to 2015, the annual average temperature in Tianjin rose from 12.6 °C to 13.6 °C^50,51^. And less or more land category might be fulfilling a certain climate target^52^. So for the sake of the future generation, urban planning needs to be conducted with higher sustainability awareness.

In this paper, there are two limitations. First, the land use intensity analysis was used to analyze the changes of land use in detail, but the impact of these changes on human society, natural ecological environment and other aspects still need to be further studied. Second, the remote sensing data of each year used in this paper were not collected on the same date, and the effect of precipitation on data quality may lead to the error for water area. So how to reduce the impact of different daily precipitation on the images also deserves further study.

## Conclusions

In this paper, new models were developed to describe change intensity of both gain category and loss category of land use from different perspectives. The new models quantified the contribution of one land category to other land categories and the contribution of all other land categories to a specific land category. The land use change was described in a more comprehensive way, and the theory of land use intensity was applied completely and meaningfully. And then the improved theory was applied to the LULC in Tianjin from 1995 to 2015. It provided a reference for the sustainable development of land use in Tianjin.

In general, agricultural land, water and urban land accounted for majority of land in Tianjin. During the two decades in this study, agricultural land and urban land changed most, with area of agricultural land reduced by about 7.5% and that of urban land increased by about 7.5%. Area of forest, water and other land all increased slightly.

As for the intensity and stationarity analysis of land use change at the interval level, transition intensity of land use during 1995–2000 was greater than the average intensity, implying a stable state; while transition intensity of land use during 2000–2005, 2005–2010 and 2010–2015 were smaller than the average intensity, implying an unstable state. At the category level, during four time periods of the study, change intensity of water, urban land and other land were all greater than the average intensity, implying being active in change; change intensity of forest and agricultural land were less than the average intensity, implying being dormant in change; change of all land use categories were stable. At the transition level, agricultural land accounted for 85% of land which was transformed to urban land. However, this represented only 45% of all agricultural land loss. The other main transition of agricultural land was water and it was also the main source of forest land. As for gain land use urban land, all transitions from agricultural land were stable. And for loss land use agricultural land, all transitions to urban land were stable, and to other land were unstable.

## Materials

### Study area

**Figure 6:**
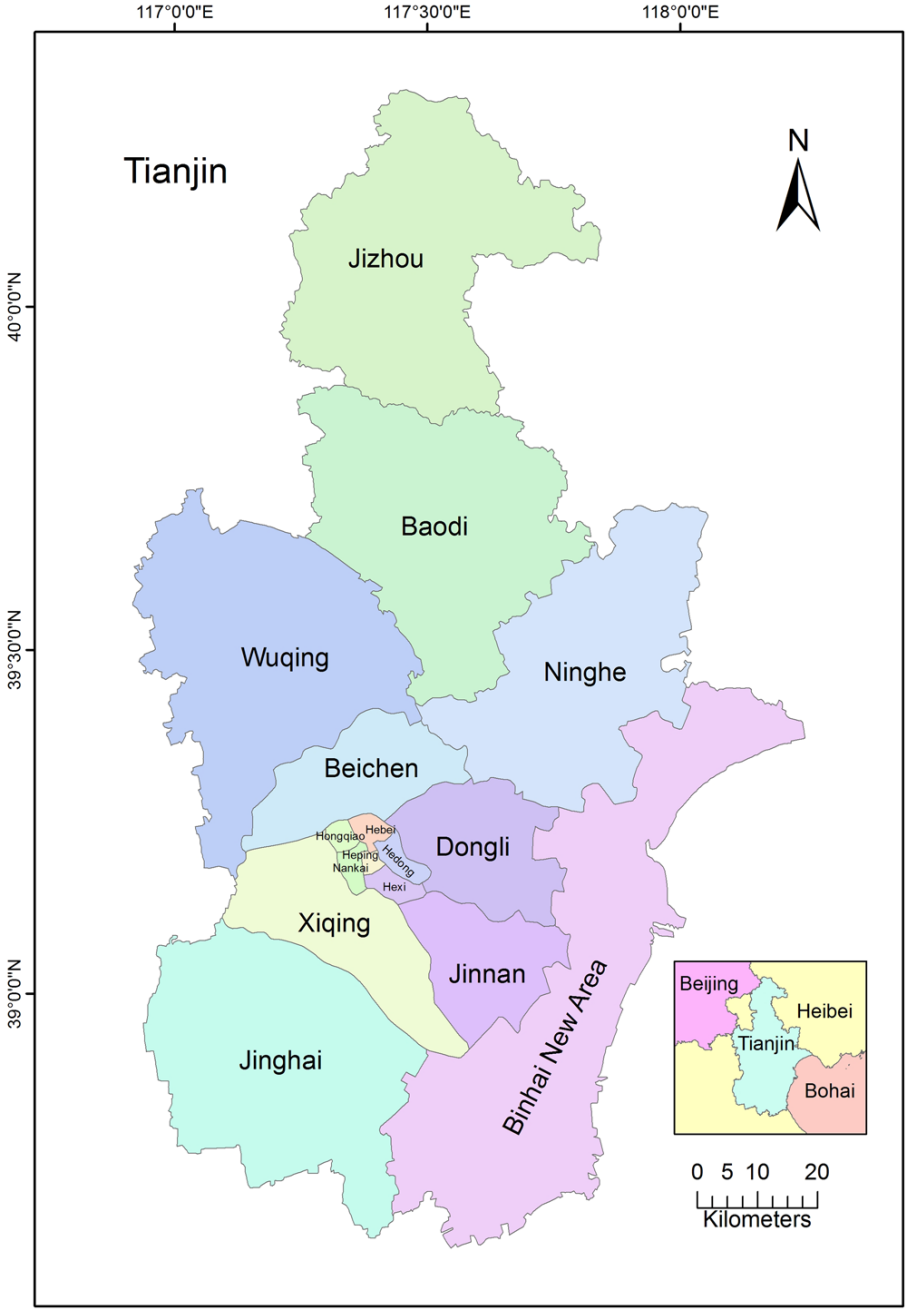
Geographic position of the study area.

Tianjin is located in northern part of the North China Plain, with its east neighboring the Bohai Sea and north close to Yanshan (Fig. 6). Tianjin has warm temperate semi-humid monsoon climate, and within the area covering 116°43′–118°04′E longitude and 38°34′–40°15′N latitude, it is affected seriously by marine climate. Four seasons are distinct in Tianjin: climate is windy, dry and rainless in spring; it is hot and rainy in summer; it is cool and mesothermal in autumn; it is cold, dry and snowless in winter. The annual average temperature is about 14 °C. The hottest month is July, with average monthly temperature reaching 28 °C and the highest temperature reaching 41.6 °C. The coldest month is January, with average monthly temperature of −2 °C and the lowest temperature of −17.8 °C. The average annual precipitation in Tianjin is 360–970 mm. Tianjin is a municipality city, economic center of the Bohai Sea areas, and national shipping center of the north world.

### Data

In this paper, the data used were TM remote sensing images in 1995, 2000, 2005, 2010, and OLI remote sensing image in 2015, which all covered Tianjin. It also included administrative map and other textual data. The remote sensing data were downloaded from the USA Geological Survey (http://glovis.usgs.gov), and the spatial coordinate system was WGS_1984_UTM_50N. Administrative map was downloaded from Geographical Information Monitoring Cloud Platform (http://www.dsac.cn/DataProduct/Index/201932). The textual data were collected from the web of Tianjin Statistics Bureau (http://stats.tj.gov.cn). The principles of image selection are as follow: (1) The period when vegetation growth could be detected since forest and agricultural land are important land use categories in the study area; (2) All images must be high-quality with value of maximum cloud less than 10%; (3) For Tianjin, four images are need to mosaic together so that images are selected with similar time as much as possible after excluding images with poor quality. Based on these principles, all images were acquired from April to September.

## Methods

### Image preprocessing

The first step was radiometric calibration that converted the DN value of image into a radiance value. The second step was atmospheric correction that eliminated radiation errors caused by atmospheric scattering and absorption in remote sensing images^53^. The third step was image merge that merged all individual bands. TM images were obtained by merging all bands and OLI image was derived by merging 2–7 bands. The fourth step was image mosaic that combined multi-view adjacent remote sensing images into a large, seamless image. Histogram matching was chosen to do color correction. Auto generate seamlines were chosen using ENVI. Cubic convolution was chosen on resampling method. The last step was image clip that obtained images of the study area using Tianjin administrative map.

### Image classification and accuracy assessment

According to the national standard *Current Land Use Classification* (GB/T 21010-2017), spatial resolution of Landsat images and regional characteristics of Tianjin, land use in the study area was divided into five categories: forest, agricultural land, urban land, water and other land. The forest refers to land where trees, bamboos and shrubs are grown, and does not include greening land within the towns and villages. The agricultural land refer to the land on which crops are grown, including paddy fields, irrigated land, and fallow fields. The urban land refers to residential land, transportation land, commercial service land and public management service land. The water refers to inland waters, tidal flats, ditches, swamps, and hydraulic structures. The other land refers to land other than the above-mentioned land. The images were classified and land use information was extracted using CART^54^.

The CART algorithm is one of the algorithms for generating decision trees. It simply divides a sample into two sub-samples so that each non-leaf node has two branches. A decision tree generated by this algorithm is referred to as a binary tree. Gini is used as split node which measure impurity at split. The range of Gini is from 0 to 1 and the larger Gini the greater the degree of unevenness in the sample set^55^. The following is the process of land use classification based on the CART algorithm. First, the multivariate dataset was built. The classification dataset included seven multi-spectral bands in TM/OLI image, NDVI and the result of ISODATA unsupervised classification with the five categories mentioned before. The ISODATA unsupervised classification algorithm is a clustering method. When the distance between two types of cluster centers is less than a certain threshold, they are combined into one class. When a certain standard deviation is greater than a certain threshold or the number of samples exceeds a certain threshold, it is divided into two categories. When the number of samples in a certain class is less than a certain threshold, it needs to be canceled. In this way, according to the initial clustering center and the number of categories set, iteratively, a better classification result is finally obtained. Second, rule was acquired. The decision tree was obtained by training samples. And training samples were acquired by uniformly drawing for each category in the remote sensing images. Third, land use information was extracted. Land use information was extracted through choosing the decision tree which was obtained from the second step. Fourth, post-processing was implemented based on the classification map from the third step. And post-processing included merging adjacent similar classification areas, filtering too small classification areas and modifying the wrong classification boundary. At last, land use classification was obtained after correcting the incorrect image boundary by visual interpretation.

The accuracy of remote sensing image was assessed by accuracy of producers and users, overall classification accuracy and kappa coefficient which was calculated by confusion matrix. Producer accuracy is the number of pixels that are correctly classified in each class as a percentage of the pixels that the class uses as a training sample. User accuracy is the number of pixels that are correctly classified for each category as a percentage of the total number of pixels that are classified as the class. Overall classification accuracy is the percentage of the total number of pixels correctly classified to the total number of pixels. Kappa coefficient is defined by Eq. 1. The number of random points used as accuracy assessment for each image was 250.

1 $$\mathrm{Kappa}\,\,{\rm{coefficient}}=\frac{N{\sum }_{i}^{m}{X}_{ii}-{\sum }_{i}^{m}({X}_{i+}{X}_{+i})}{{N}^{2}-{\sum }_{i}^{m}({X}_{i+}{X}_{+i})}$$

In Eq. 1, N denotes the total number of pixels used for accuracy evaluation, m denotes the number of rows in the confusion matrix, X_ii_ is the number of pixels on the i-th row in the i-th column, X_i+_ is the total number of pixels in the i-th row, X_+i_ is the total number of pixels in the i-th column.

### Intensity and stationarity analysis of land use change

The intensity and stationarity analysis of land use change focus on the change itself. Intensity analysis as a top-down hierarchical accounting framework includes interval level, category level and transition level^42^.

Analysis at interval level reflects total change in each time interval. Intensity analysis uses land use change intensity S(t) (Eq. 2) and uniform intensity U (Eq. 3) to explain land use change during a certain period^42^. If S(t) is greater than U, the change of land use is fast; vice versa. Stationarity analysis in each time interval is carried out through comparing S(t) and U. If the S(t) is evenly distributed over the whole time interval, the change is stable. Otherwise, it is unstable.

2 $${\rm{S}}({\rm{t}})=\frac{\{{\sum }_{{\rm{j}}=1}^{{\rm{J}}}[({\sum }_{{\rm{i}}=1}^{{\rm{J}}}{{\rm{C}}}_{{\rm{tij}}})-{{\rm{C}}}_{{\rm{tjj}}}]\}/[{\sum }_{{\rm{j}}=1}^{{\rm{J}}}({\sum }_{{\rm{i}}=1}^{{\rm{J}}}{{\rm{C}}}_{{\rm{tij}}})]}{{{\rm{Y}}}_{{\rm{t}}+1}-{{\rm{Y}}}_{{\rm{t}}}}\ast 100 \% $$

3 $${\rm{U}}=\frac{{\sum }_{{\rm{t}}=1}^{{\rm{T}}-1}\{{\sum }_{{\rm{j}}=1}^{{\rm{J}}}[({\sum }_{{\rm{i}}=1}^{{\rm{J}}}{{\rm{C}}}_{{\rm{tij}}})-{{\rm{C}}}_{{\rm{tjj}}}]\}/[{\sum }_{{\rm{j}}=1}^{{\rm{J}}}({\sum }_{{\rm{i}}=1}^{{\rm{J}}}{{\rm{C}}}_{{\rm{tij}}})]}{{{\rm{Y}}}_{{\rm{T}}}-{{\rm{Y}}}_{1}}\ast 100 \% $$

In Eqs 2–3, i and j denote the number of land categories, Ctij denotes the area that transformed from category i to category j at the time t, Yt is time.

Analysis at the category level studies the intensity change of each land category within a particular time interval. It uses annual gain intensity of Gtj (Eq. 4) and annual loss intensity Lti (Eq. 5) to explain land use change of a certain land category^42^. If Gtj or Lti is greater than U, the change of this land category is active; otherwise, it is dormant. Stationarity analysis for each land category is conducted through comparing Gtj, Lti and U: if the Gtj or Lti is evenly distributed in the whole space, the change is stable; otherwise, it is unstable.

4 $${{\rm{G}}}_{{\rm{tj}}}=\frac{[({\sum }_{{\rm{i}}=1}^{{\rm{J}}}{{\rm{C}}}_{{\rm{tij}}})-{{\rm{C}}}_{{\rm{tjj}}}]/({{\rm{Y}}}_{{\rm{t}}+1}-{{\rm{Y}}}_{{\rm{t}}})}{{\sum }_{{\rm{i}}=1}^{{\rm{J}}}{{\rm{C}}}_{{\rm{tij}}}}\ast 100 \% $$

5 $${{\rm{L}}}_{{\rm{ti}}}=\frac{[({\sum }_{{\rm{j}}=1}^{{\rm{J}}}{{\rm{C}}}_{{\rm{tij}}})-{{\rm{C}}}_{{\rm{tii}}}]/({{\rm{Y}}}_{{\rm{t}}+1}-{{\rm{Y}}}_{{\rm{t}}})}{{\sum }_{{\rm{j}}=1}^{{\rm{J}}}{{\rm{C}}}_{{\rm{tij}}}}\ast 100 \% $$

Analysis at the transition level studies the transition of a particular land category. As for land category n, Rtin (Eq. 6) is the transition intensity from category i to category n in a particular time interval t; and Wtn is the average transition intensity during this time interval (Eq. 7), with assumption that the land category n is increasing over the entire time period^42^. As for land category m, Qtmj (Eq. 8) is the transition intensity from category m to category j in a particular time interval, and Vtm is the average transition intensity during this time interval (Eq. 9), with assumption that the land category m is decreasing over the entire time period^42^.

The transition intensity of each land category is compared with the uniform intensity. If the transition intensity is evenly distributed over the whole space, the change is stable. Otherwise, it is unstable.

6 $${{\rm{R}}}_{{\rm{tin}}}=\frac{{{\rm{C}}}_{{\rm{tin}}}/({{\rm{Y}}}_{{\rm{t}}+1}-{{\rm{Y}}}_{{\rm{t}}})}{{\sum }_{{\rm{j}}=1}^{{\rm{J}}}{{\rm{C}}}_{{\rm{tij}}}}\ast 100 \% $$

7 $${{\rm{W}}}_{{\rm{tn}}}=\frac{[({\sum }_{{\rm{i}}=1}^{{\rm{J}}}{{\rm{C}}}_{{\rm{tin}}})-{{\rm{C}}}_{{\rm{tnn}}}]/({{\rm{Y}}}_{{\rm{t}}+1}-{{\rm{Y}}}_{{\rm{t}}})}{{\sum }_{{\rm{j}}=1}^{{\rm{J}}}[({\sum }_{{\rm{i}}=1}^{{\rm{J}}}{{\rm{C}}}_{{\rm{tij}}})-{{\rm{C}}}_{{\rm{tnj}}}]}\ast 100 \% $$

8 $${{\rm{Q}}}_{{\rm{tmj}}}=\frac{{{\rm{C}}}_{{\rm{tmj}}}/({{\rm{Y}}}_{{\rm{t}}+1}-{{\rm{Y}}}_{{\rm{t}}})}{{\sum }_{{\rm{i}}=1}^{{\rm{J}}}{{\rm{C}}}_{{\rm{tij}}}}\ast 100 \% \,$$

9 $${{\rm{V}}}_{{\rm{tm}}}=\frac{[({\sum }_{{\rm{j}}=1}^{{\rm{J}}}{{\rm{C}}}_{{\rm{tmj}}})-{{\rm{C}}}_{{\rm{tmm}}}]/({{\rm{Y}}}_{{\rm{t}}+1}-{{\rm{Y}}}_{{\rm{t}}})}{{\sum }_{{\rm{i}}=1}^{{\rm{J}}}[({\sum }_{{\rm{j}}=1}^{{\rm{J}}}{{\rm{C}}}_{{\rm{tij}}})-{{\rm{C}}}_{{\rm{tim}}}]}\ast 100 \% $$

In this paper, Ptin (n), Ptin (i), Otmj(m) and Otmj (j) were defined and new models were developed at transition level to describe land use change situations in more detail. Ptin (n) (Eq. 10) is used to describe change intensity from land category i to category n in a certain time interval from perspective of gain land category n; Ptin (i) (Eq. 11) is used to describe that from perspective of land category i. Otmj (m) (Eq. 12) is used to describe change intensity from land category m to category j in a certain time interval from perspective of loss category m; Otmj (j) (Eq. 13) is used to describe that from perspective of changed category j.

10 $${{\rm{P}}}_{{\rm{tin}}({\rm{n}})}=\frac{{{\rm{C}}}_{{\rm{tin}}}}{{\sum }_{{\rm{i}}=1}^{{\rm{J}}}{{\rm{C}}}_{{\rm{tin}}}}\ast 100 \% $$

11 $${{\rm{P}}}_{{\rm{tin}}({\rm{i}})}=\frac{{{\rm{C}}}_{{\rm{tin}}}}{{\sum }_{{\rm{j}}=1}^{{\rm{J}}}{{\rm{C}}}_{{\rm{tij}}}}\ast 100 \% $$

12 $${{\rm{O}}}_{{\rm{tmj}}({\rm{m}})}=\frac{{{\rm{C}}}_{{\rm{tmj}}}}{{\sum }_{{\rm{j}}=1}^{{\rm{J}}}{{\rm{C}}}_{{\rm{tmj}}}}\ast 100 \% $$

13 $${{\rm{O}}}_{{\rm{tmj}}({\rm{j}})}=\frac{{{\rm{C}}}_{{\rm{tmj}}}}{{\sum }_{{\rm{i}}=1}^{{\rm{J}}}{{\rm{C}}}_{{\rm{tij}}}-{{\rm{C}}}_{{\rm{tjj}}}}\ast 100 \% $$

## Data Availability

The Landsat data are publicly available here: http://glovis.usgs.gov. The administrative maps are publicly available here: http://www.dsac.cn/DataProduct/Index/201932. The textual data are publicly available here: http://stats.tj.gov.cn.
